# Characterization of the *GGP* gene family in potato (*Solanum tuberosum* L.) and Pepper (*Capsicum annuum* L.) and its expression analysis under hormonal and abiotic stresses

**DOI:** 10.1038/s41598-024-66337-x

**Published:** 2024-07-03

**Authors:** Zhiqi Ding, Kangding Yao, Yandong Yao, Xuejuan Pan, Lizhen Luo, Long Li, Chunlei Wang, Weibiao Liao

**Affiliations:** College of Horticulture, Gansu Agricultural University, Lanzhou, 730070 People’s Republic of China

**Keywords:** Abiotic stress, Ascorbic acid, *Capsicum annuum* L, Gene family, *GGP*, Phytohormone, *Solanum tuberosum* L, Genetics, Plant sciences

## Abstract

GDP-l-galactose phosphorylase (GGP) is a key rate-limiting enzyme in plant ascorbic acid synthesis, which plays an important role in plant growth and development as well as stress response. However, the presence of *GGP* and its function in potato and pepper are not known. In this study, we first identified two *GGP* genes in each potato and pepper genomes using a genome-wide search approach. We then analyzed their physicochemical properties, conserved domains, protein structures and phylogenetic relationships. Phylogenetic tree analysis revealed that members of the potato and pepper *GGP* gene families are related to eggplant* (Solanum melongena* L.*)*, Arabidopsis (*Arabidopsis thaliana* L.), tobacco (*Nicotiana tabacum* L.) and tomato (*Solanum lycopersicum* L.), with tomato being the most closely related. The promoter sequences mainly contain homeopathic elements such as light-responsive, hormone-responsive and stress-responsive, with light-responsive elements being the most abundant. By analyzing the structure of the genes, it was found that there is no transmembrane structure or signal peptide in the *GGP* gene family of potatoes and peppers, and that all of its members are hydrophilic proteins. The expression profiles of different tissues show that *StGGP1* has the highest expression levels in leaves, *StGGP2* has the highest expression levels in stamens, and *CaGGPs* have the highest expression levels in the early stages of fruit development (Dev1). It was found that *StGGPs* and *CaGGPs* genes showed different response to phytohormones and abiotic stresses. Abscisic acid (ABA) treatment induced the most significant change in the expression of *StGGPs*, while the expression of *CaGGPs* showed the most pronounced change under methyl jasmonate (MeJA) treatment. *StGGPs* responded mainly to dark treatment, whereas *CaGGPs* responded mainly to NaCl stress. These results provide an important basis for a detailed study about the functions of *GGP* homologous genes in potato and pepper in response to abiotic stresses.

## Introduction

Ascorbic acid (AsA) known as vitamin C is the most abundant water-soluble antioxidant in plant cells. It plays an important role in photosynthesis and stress resistance, and is involved in epigenetic activation^1^. Ascorbate mitigates the harmful effects of reactive oxygen species (ROS) produced by normal or stressed cellular metabolism, either directly as a ROS scavenger or indirectly as a substrate for the enzyme ascorbate peroxidase^2^. In addition, AsA helps plants to resist adverse environmental conditions, and it has been shown in wheat (*Triticum aestivum* L.) that ascorbic acid could significantly alleviate salt stress and drought stress^3^. The exogenous application of AsA significantly increased the activities of peroxidase (POD), catalase (CAT) and superoxide dismutase (SOD) and reduced the Pb-induced oxidative damage in okra under Pb stress. Exogenous AsA played a similar role in alleviating stress damage in rice (*Oryza sativa* L.) under aluminum stress^4^. AsA also increases the activity of antioxidant enzymes, photosynthesis capacity and the maintenance of salt stress tolerance in wheat, tomatoes (*Solanum lycopersicum* L.), strawberries (*Fragaria ananassa Duch.*) and cowpeas (*Vigna unguiculata* L.), thus counteracting the adverse effects of salt stress on plant growth^5–8^. There are four pathways used to synthesize ascorbic acid in plants: the l-galactose, l-glutathione, inositol and d-galacturonic acid pathways^9^. The l-galactose pathway is the main pathway for ascorbate biosynthesis in Arabidopsis (*Arabidopsis thaliana* L.) and tomato^10^. GDP-l-galactose phosphorylase (GGP), also known as GDP-l-galactosyl transferase (GGT), catalyzes the conversion of GDP-l-galactose to l-galactose-1-phosphate and is considered a key control node in the AsA biosynthesis pathway^11^. In addition, *GGPase* has been shown to be the rate-limiting step in the l-galactose pathway^12^.

*GGP* is the gene for GDP-l-galactose phosphorylase, which catalyzes the conversion of GDP-l-galactose to l-galactose-1-phosphate, and is the last gene cloned in the d-mannose/l-galactose pathway, the main pathway of ascorbate synthesis. The *GGP* gene plays an important role in the synthesis and regulation of ascorbic acid. It has been studied in crops such as wheat, maize (*Zea mays* L.) and tomato^13^. It has been found that *SlGGP* was significantly reduced in transcript abundance in *slgme2* overexpressing plants, suggesting that GGP may play an important role in tomato ascorbic acid synthesis^14^. It has been previously shown that AsA levels in tomato were mainly controlled by post-transcriptional repression of GGP, and down-regulation of *GGP* resulted in a nearly 1.5-fold reduction in leaf AsA content, highlighting the dominant role of *GGP* in regulating AsA levels in tomato^15^. The banana (*Musa acuminata*) genome database identified five *GGPs*^16^. Both rice and maize have a single copy of the *GGP* gene in their genomes, which is located on the Os11 and Zm6 chromosomes, respectively^9^. The expression of the *GGP* gene showed a good correlation with the corresponding ascorbic acid content in leaves and fruits of kiwifruit (*Actinidia chinensis*) at different development stages^17^. GGP proteins not only help to anabolize ascorbate but may also act as growth regulators. In Arabidopsis vtc2 mutants, VTC2: GUS fusion proteins were found in both cytoplasmic and nuclear locations, indicating that VTC2 might play a catalytic role in cytoplasm and a specific role in nuclear gene expression regulation^18^.

Both potato (*Solanum tuberosum* L.) and pepper (*Capsicum annuum* L.) belong to the Solanaceae family, which are important crops with significant commercial and nutritional value. Potato is the third most important food crop globally, known for its adaptable growing habit, allowing it to thrive in various climatic and soil conditions^19^. It is grown and consumed worldwide. Additionaly, pepper, a common vegetable crop, is more adaptable to warmer climates, requiring more heat and sunlight to grow^20^. Although the potato and pepper are both members of the nightshade family, they have very different morphology, growing conditions and use. As they grow in different environments and may be exposed to different biotic and abiotic stresses, their tolerance and stress resistance may also differ. At the genomic level, however, both potatoes and pepper possess the *GGP* gene family. This is a key gene for the synthesis of ascorbic acid, which plays a crucial role in the growth, development and resistance of plants. Therefore, studying the *GGP* gene families of potatoes and peppers may not only help us understand how these two crops adapt to their respective environments by regulating ascorbic acid synthesis, but also provide new strategies for crop variety improvement and increasing the nutritional value of crops. Many studies on potato and pepper gene families have been reported with the availability of the Solanaceae genome database. However, knowledge of the *GGP* family in potatoes and pepper is very limited. Therefore, to better understand the key role of the GGP family in plants, the coding genes of potato and pepper GGP family members were characterized and analyzed in this study. Members of the potato and pepper *GGPs* were analyzed for gene structure, secondary structure, chromosomal localization, conserved motif analysis, cis-acting element analysis, phylogenetic tree and subcellular localization. In addition, the expression patterns of these *GGPs* in different tissue-specific processes were investigated, as well as gene transcription analysis under different abiotic stresses and hormonal conditions. This study was designed to lay the groundwork for future research into the role of *GGPs* in growing, developing and resisting in potato and pepper.

## Results

### Identification of members of the *GGP* gene family in potato and pepper

**Figure 1:**
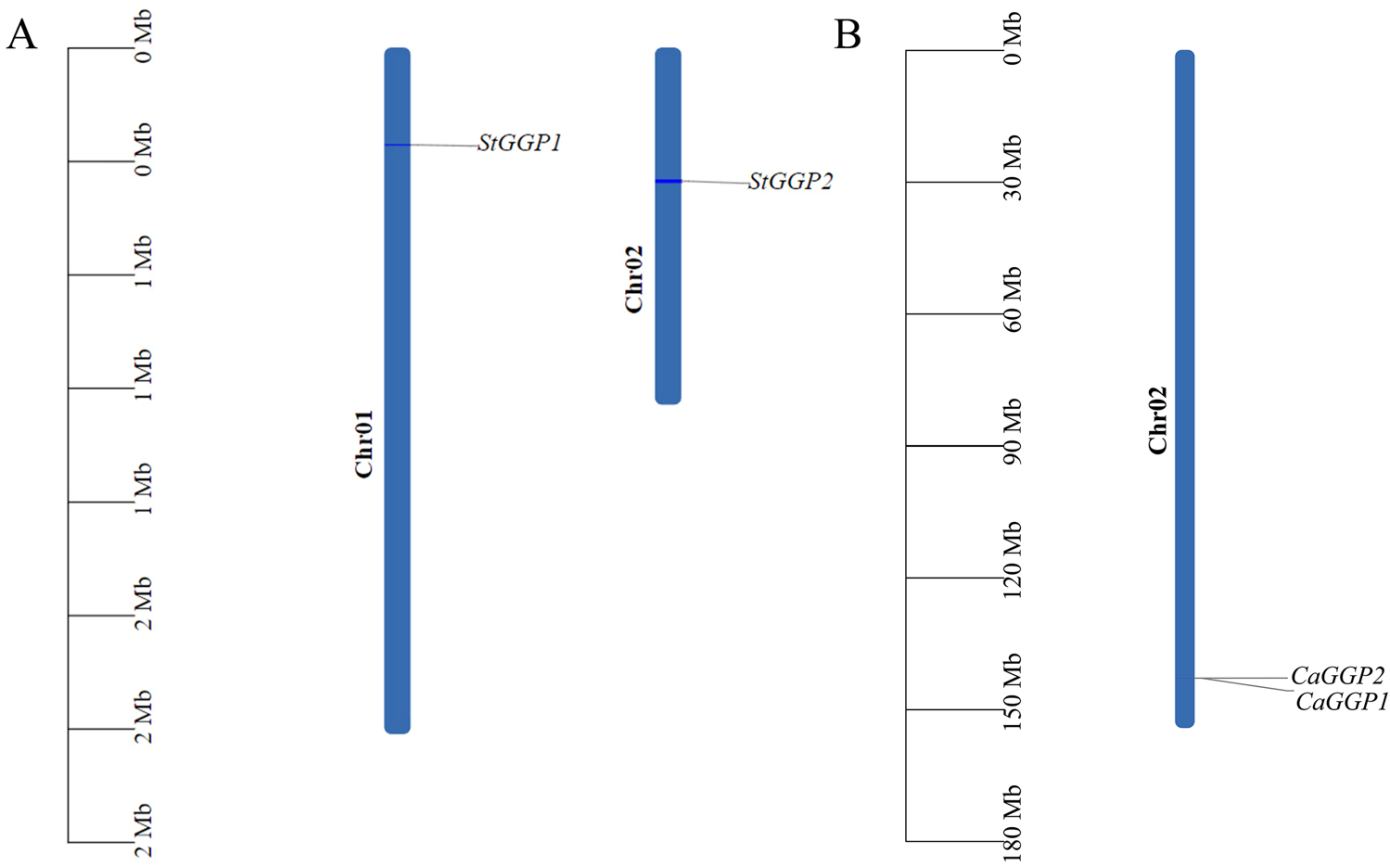
Chromosomal location of the *GGP* gene. (**A**) Chromosomal location of the potato *GGP* gene, (**B**) chromosomal location of the pepper *GGP* gene, chromosomal numbers shown at top of each bar. Gene names are shown in black. Scale bars are shown on the left.

A total of four (two for potato and two for pepper) protein sequences containing the GGP structural domain were retrieved from the potato and pepper genome databases (E-value = 1e–5). Screening removed incomplete proteins and proteins whose annotation information in Blastp (database SSWISS-PROT/Uniprot) in NCBI are not a GGP structural domain, resulting in the identification of four (two each for potato and pepper) *GGP* gene (Table 1). Based on their chromosomal distribution, potatoes were sequentially named StGGP1 and *StGGP2*, and pepper, *CaGGP1* and *CaGGP2*. The distribution of *GGP* genes on chromosomes was visualized using the genome annotation files of potato and pepper. The potato *GGP* genes are unevenly distributed across two chromosomes, and the pepper *GGP* genes are located on the same chromosome, as shown in Fig. 1. The number of genes on each chromosome is independent of the size of the chromosome.

**Table 1:** Identification of potatoes and pepper *GGP* gene family.

Gene	Gene ID	Genome matching section/bp	The positive and negative chain
*StGGP1*	102596542	254672–257608	−
*StGGP2*	102593176	347521–356656	+
*CaGGP1*	107860116	142822364–142828691	−
*CaGGP2*	107860116	142822746–142828631	−

### Analysis of conserved structural domains and gene structure of *GGP* family members in potato and pepper

**Figure 2:**
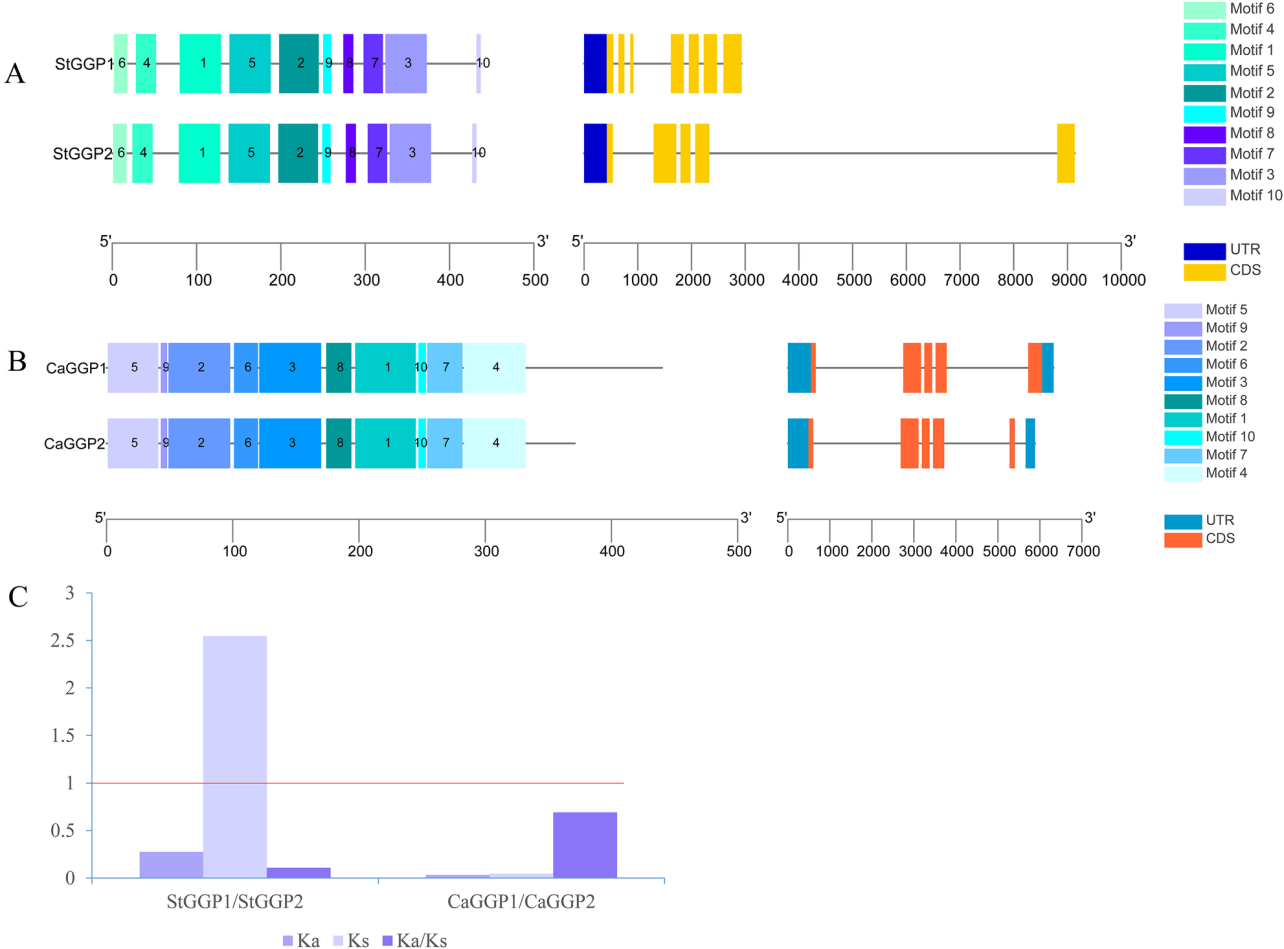
Motif, gene structure and selective evolutionary pressures in the potato and pepper *GGP* gene families. (**A**) motif and gene structure of the potato *GGP* gene family; (**B**) motif and gene structure of the pepper *GGP* gene family; on the left is the motif, with different colors representing different motifs; on the right is the gene structure; (**C**) selective evolutionary pressures (Ka/Ks).

Figure 2 shows the conserved motifs of the *GGP* gene family and visualization of evolutionary relationships, conserved structures and gene structures of potato and pepper *GGP* genes. The conserved structural domains showed that both potato and pepper *GGP* families contained 10 conserved motifs, and all members contained identical conserved motifs (Motif), i.e., Motif 1–Motif 10, and the specific sequence information is shown in Table 2. The structural analysis of the genes showed that the *GGP* genes of the pepper all contain five exons, in addition to *CaGGP1*, which has four introns, and *CaGGP2*, which has five introns.

**Table 2:** Potato and pepper *GGP* motif information.

Species	Motif	Motif sequence	Length (AA)	Functional motif	Domains	pfam numbers
Potato	Motif1	WEERQQKGLFRYDVTACETKVIPGECGFIAQLNEGRHLKKRPTEFCIDKV	50	Yes	cl47078	IPR026506
	Motif2	CLPQRIDRDSFAJALHFAREVADPFFRLGYNSLGAFATINHLHFQAYF	48	Yes		
	Motif3	PQCYAEKQALGEVDQELLDTQVNPAVWEISGHIVLKRKEDYEGASEENAW	50	No		
	Motif4	AGCGRKCLGKCCLPGSKLPLYGFKN	25	No		
	Motif5	NFTKVGQEELLFRFEPSEEDEPQLFPGARIDGEKSPSIVAINVSPIEYGH	50	Yes		
	Motif6	MLKIKRVPTLVSNFQED	17	Yes		
	Motif7	SCICLQEKNIPFNILIADCGKKIF	24	No		
	Motif8	NYPVRGFVFEGGN	13	No		
	Motif9	FPIEKAPIQKI	11	Yes		
	Motif10	ASHMPP	6	No		
Pepper	Motif1	DCLPQRIGRDTFTIALHFAREMADPFFRVGYNSLGAFATINHLHYQAYY	49	Yes	cl47078	IPR026506
	Motif2	NDDNEPMEHNIHTLPEEEYQISFLNNLLLGLWEERMSQGLFRYDVTTCET	50	Yes		
	Motif3	PTEFRIDKVLQPFDENKFNFTKVGQDEVLFRFEPSTDYKRRYFSGMGVDA	50	No		
	Motif4	GFTFEGENGSTIRDLSEVVVNSCISLQNKNIPFNILIAQCGKKIFLFPQC	50	Yes		
	Motif5	MLTIKRVPTVVSNYQEDVLLESNVVGCGRKCLGKCCLPVSM	41	Yes		
	Motif6	IPGKCGFIAQLNEGRHLKKR	20	Yes		
	Motif7	KAPVRKILARKGLGGAGVIVSKLLNYPVR	29	No		
	Motif8	PSIVAINVSPIEYGHVLLIPR	21	No		
	Motif9	PLYSFK	6	No		
	Motif10	SVPFPVE	7	No		

However, the gene structures of the potato *GGPs* are very different. *StGGP1* has seven exons and six introns, while *StGGP2* has five exons, four introns and a longer intron. In addition, the values of the selective evolutionary pressure showed that both the *GGP* genes of the potato and the pepper were calculated to be less than 1 and that both had a tendency to be purified (Fig. 2C).

### Multiple series comparison and phylogenetic analysis of the *GGP* family of potato and pepper

**Figure 3:**
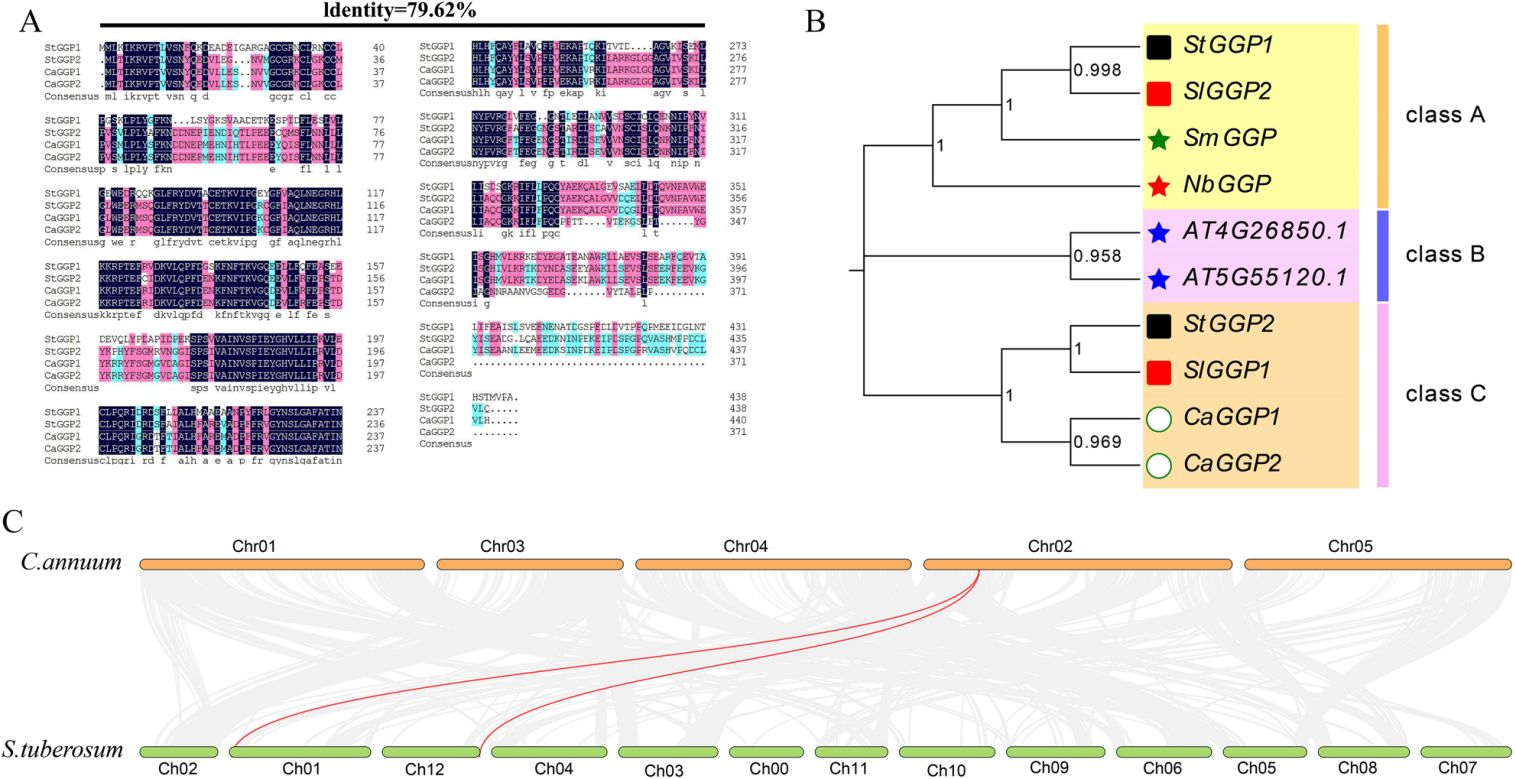
Multiple sequence comparisons, collinearity analysis, and phylogenetic trees of the potato, pepper, tomato, eggplant, Arabidopsis, and tobacco *GGP* gene families. (**A**) Multiple sequence comparison of potato and pepper *GGPs*. (**B**) Phylogenetic trees of the *GGP* families of potatoes, pepper, tomatoes, eggplants, Arabidopsis and tobacco. The six different species are represented by the six different colored shapes. The red pentagram represents tobacco. The blue pentagram represents Arabidopsis thaliana. The green pentagram represent eggplant. The black squares represent potatoes. The red squares represent tomatoes. The white circles represent pepper. (**C**) The collinearity analysis of the *GGP* gene family was conducted in pepper and potato. Syntenic *GGP* gene pairs are represented by the marked red lines.

The protein sequences of the potato and pepper GGP families have been analyzed. The most conserved sequences are annotated in dark blue, followed by red and blue. As shown in Fig. 3A, the homology between members of the potato and pepper *GGP* families was as high as 79.62%, indicating that the family is evolutionarily conserved.

To study the evolutionary relationship of potato and pepper *GGPs* with *GGPs* of the template crop *Arabidopsis thaliana* and other species, a phylogenetic tree of GGP protein sequences in Arabidopsis, tomato, eggplant, tobacco, potato, and pepper was constructed. The results showed that the 10 GGP proteins from these plants were categorized into three subgroups (A, B, and C), with *StGGP2* and *SlGGP1* being the most closely related subgroup (four members). Class B includes two members, and both compose of Arabidopsis GGP proteins. In addition, tomato is the most closely related to potato among the three subgroups, suggesting an evolutionary link between potato and tomato. Meanwhile, the *GGP* family members of potato and pepper are closely related to those of eggplant, Arabidopsis, and tomato (Fig. 3B). The results of collinearity analysis showed that two pepper genes are collinear with two potato genes, there are two homologous pairs between pepper and potato (Fig. 3C).

### Analysis of the role of cis-elements of potato and pepper *GGP* genes

**Figure 4:**
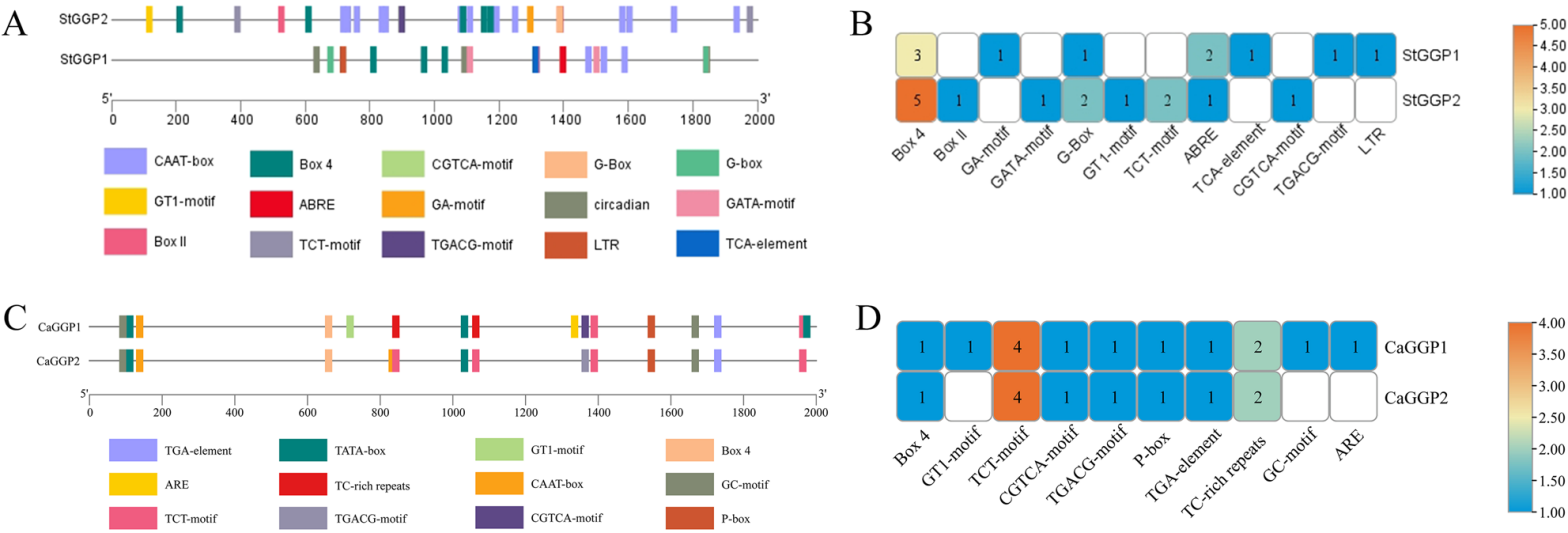
Analysis of cis-acting elements in potato and pepper. (**A**) Potato homeopathic component visualization. (**B**) Potato homeopathic component type statistics. (**C**) Pepper homeopathic component visualization. (**D**) Pepper homeopathic component type statistics. Different colored wedges represent different cis elements. The length and position of each *GGP* gene were mapped to scale. The scale bar represents the length of the DNA sequence. The length and position of each *GGP* gene were mapped to scale. The scale bar represents the length of the DNA sequence.

In order to understand the expression regulation of potato and pepper *GGP* genes, 2000 bp upstream of the potato and pepper *GGP* genomes were extracted and submitted to PlantCARE for promoter analysis. The potato predictions revealed seven light-responsive elements (Box 4, Box II, GA motif, GATA motif, G-box, GT1 motif and TCT motif); three hormone-responsive elements, including abscisic acid (ABRE), salicylic acid (TCA element) and (MeJA); and one stress element (LTR). The light-responsive elements were unevenly distributed in each of the *GGP* genes in potato, with *StGGP2* having the highest number of elements. The *StGGP1* contains two ABREs, the *StGGP2* contains one ABRE and the TCA element is distributed in the *StGGP1*. The MeJA response element was distributed in each potato *GGP* and the low temperature response element LTR was distributed in *StGGP1* (Fig. 4A, B).

Pepper predictions revealed the presence of three light responsive elements (Box 4, GT1-motif, TCT-motif), four hormone responsive elements containing methyl jasmonate (CGTCA-motif, TGACG-motif), gibberellin (P-box) and growth hormone (TGA-element) and three stress responsive elements (TC-rich repeats, GC-motif, ARE). The TCT motif, the MeJA, the P box and the TGA element were present in every *GGP* of pepper. The stress response element TC-rich repeats were distributed in *CaGGP1* and *CaGGP2* of pepper, and the anaerobically induced response element GC-motif, ARE, was distributed in *CaGGP1* (Fig. 4C, D).

### Structural analysis of proteins from the* GGP* gene family

The analysis of the physicochemical properties of the potato and pepper *GGP* family revealed that the length of the potato and pepper GGP proteins ranges from 371 to 438aa, and the theoretical isoelectric point pI, except CaGGP2 which is greater than seven, are distributed between 4.71 and 5.79, which belonged to the acidic proteins. The molecular mass of the GGP proteins from the potato and the pepper ranges from 41.5 to 49.7 kDa. In addition, neither the potato nor the pepper *GGP* family has a signal peptide or transmembrane structure, according to both signal peptide and transmembrane structure predictions. Meanwhile, it was predicted that both potato and pepper GGP proteins may be hydrophilic. They contain more hydrophilic peaks (Table 3).

**Table 3:** Analysis of physicochemical properties of potato and pepper *GGP*.

Gene	Length(aa)	Molecular weight	pI	Instability index	Grand average of hydropathicity	Signal peptide	Transmembrane domain
StGGP1	438	48,703.3	4.71	40.75	− 0.211	No	No
StGGP2	438	49,102.28	5.24	53.53	− 0.18	No	No
CaGGP1	440	49,674.12	5.79	47.78	− 0.192	No	No
CaGGP2	371	41,516.94	8.25	45.74	− 0.088	No	No

In addition, the potato and pepper *GGP* genes were found to be distributed mainly in chloroplasts, cytoplasm, nucleus and mitochondria by subcellular localization. However, the distribution of each member was different in different cellular fractions (Table 4).

**Table 4:** Subcellular localization of *GGP* in potato and pepper.

Gene	Chloroplast	Cytoplasm	Nuclear	Mitochondria	Plasmalemma	Extracellular matrix
*StGGP1*	5	5	2	1.5	–	–
*StGGP2*	6.5	4	1	–	1	–
*CaGGP1*	3.8	4	2	–	–	1
*CaGGP2*	5	5	–	–	1	1

**Figure 5:**
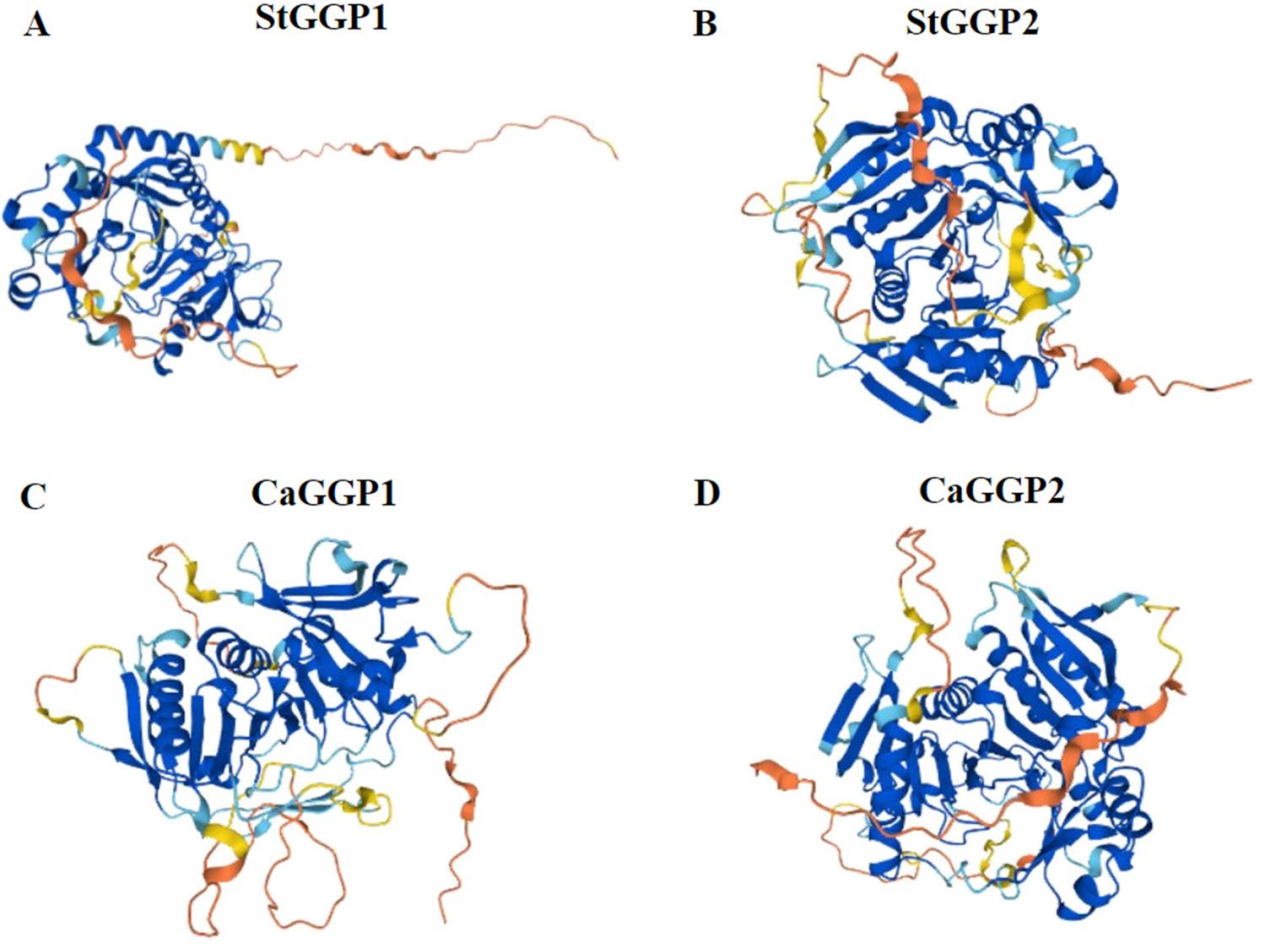
Three-stage structure model of potato and pepper *GGP*. (**A**,**B**) Tertiary structural modeling of the potato *GGP* gene family. (**C**,**D**) Tertiary structural modeling of the *GGP* gene family in pepper.

The secondary structure of the potato and pepper family *GGPs* revealed that each member of the potato and pepper family *GGPs* has the highest percentage of irregular curl (pink line) between 44.29 and 49.87%; The percentage of alpha helices (blue line) is between 22.64 and 33.33%, the percentage of elongations (red line) is between 16.67 and 22.91%, and the percentage of beta folds (green line) is the smallest, between 4.58 and 5.71% (Table 5). Based on the tertiary structure results, the structures of potato and pepper *GGP* family proteins are consistent with the structural components of the secondary predictions, and the members are modeled very similarly (Fig. 5).

**Table 5:** Distribution of secondary structural elements in potato and pepper *GGP*.

Gene	Alpha helix (%)		Beta turn (%)	Random coil (%)	Extended strand (%)		Distribution of secondary structure elements
*StGGP1*		33.33%	5.71%	44.29%	16.67%		
*StGGP2*		32.42%	4.79%	46.12%	16.67%		
*CaGGP1*		32.05%	4.77%	46.59%	16.59%		
*CaGGP2*		22.64%	4.58%	49.87%	22.91%

### Expression pattern analysis of potato and pepper *GGP* gene family in different tissues

**Figure 6:**
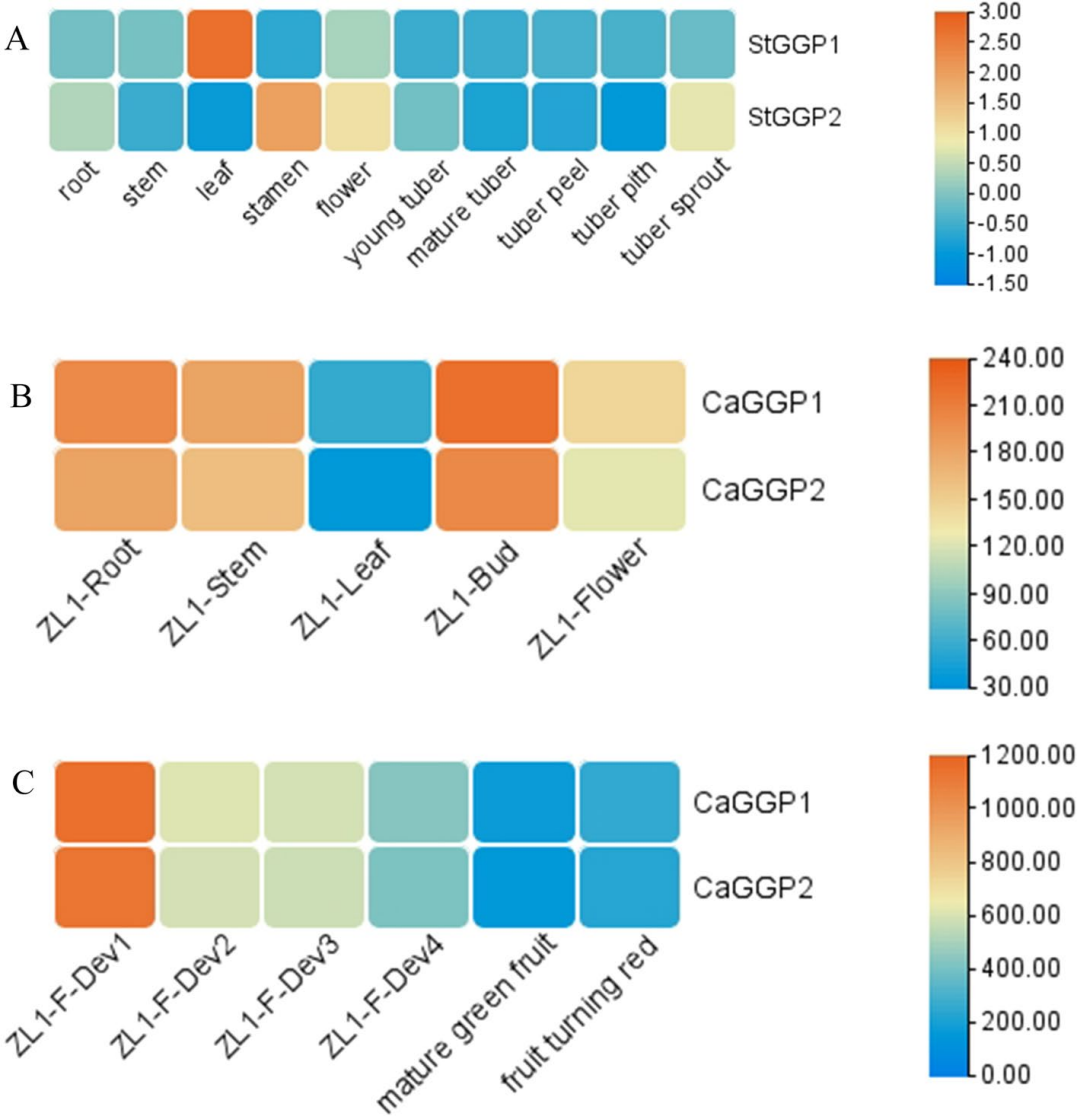
Potato data from UniPort online site (https://www.uniprot.org/), pepper data from pepper genome sequencing (https://www.ncbi.nlm.nih.gov/geo/query/acc.cgi?acc=GSE45037). Expression analysis of different tissues of potato and pepper *GGPs* gene families. (**A**) Expression pattern of potato *GGP* in different tissues. (**B**) Expression pattern of pepper *GGP* in different tissues. (**C**) *GGP* expression patterns at different times during the ripening of the fruit of the pepper. Color scale represents fold change normalized by log_2_-transformed data.

In order to confirm the tissue-specific expression patterns of *StGGPs* and *CaGGPs*, we analyzed the expression levels of the *GGPs* genes at different stages of growth and in different tissues in potato and pepper. The results showed that the expression of *StGGP1* was highest in the leaves, whereas the expression of *StGGP2* was highest in the stamens. The pepper *GGP* gene is expressed in roots, stems, leaves, flowers and fruits, with leaves showing the lowest expression. In addition, the expression of *GGPs* in pepper was highest at the beginning of fruit development (Dev1), followed by a decrease, and the lowest expression was found in mature green fruits. The expression of the pepper *GGP* gene family in different tissues and during fruit expansion was extracted from previously published data. These results indicate that the *GGPs* genes are mainly active in the leaves of the potato, whereas in the pepper, the *GGPs* are mainly active in the fruits, especially in the pre-ripening of fruits (Fig. 6).

The heatmap is shown in blue/yellow/orange colors indicating low/medium/high expression, respectively. In the figure, ZL1-F-Dev1—ZL1-F-Dev4 represents fruits with lengths of 0–1 cm, 1–3 cm, 3–4 cm and 4–5 cm.

### Expression of *GGP* gene in potato and pepper under MeJA and ABA treatments

**Figure 7:**
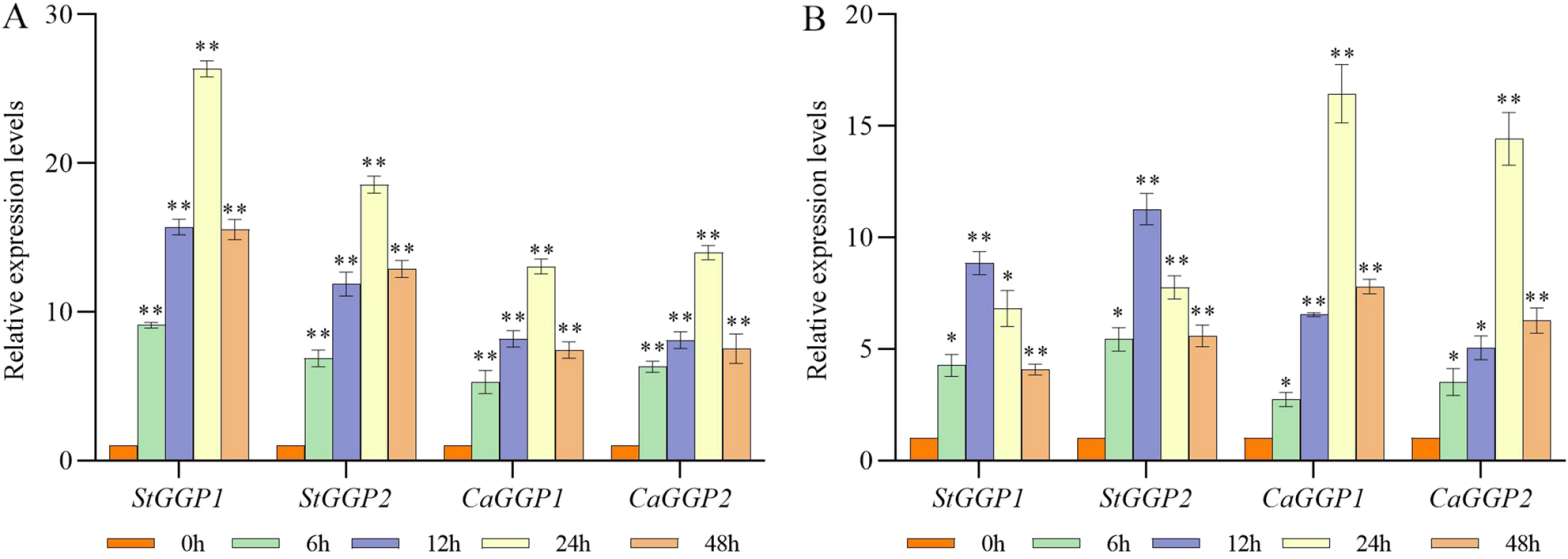
Relative expression levels of *StGGPs* and *CaGGPs* under two hormone treatments. (**A**) ABA treatment. (**B**) MeJA treatment. Expression values were normalized to the control (0 h) for each gene. An asterisk indicates a significant difference between the stress-treated and control groups. (*p* ≤ 0.05, **p ≤ 0.01, one-way ANOV A, Tukey’s test).

In order to understand the expression patterns of the *StGGPs* and *CaGGPs* genes under different hormonal treatments and abiotic stresses, we treated potato and pepper seedlings with two hormones (ABA and MeJA) as well as with four stress factors (dark, cold, NaCl and PEG). As shown in Fig. 7, the expression of *StGGP1*, *StGGP2*, *CaGGP1* and *CaGGP2* was differentially upregulated after 6 h stimulation with ABA and MeJA, and their expression levels all peaked at 24 h under ABA treatment. When treated with MeJA, *StGGP1* and *StGGP2* peaked at 12 h, whereas *CaGGP1* and *CaGGP2* peaked at 24 h, and the expression levels of the genes gradually declined after having peaked.

### Expression profiling of the *GGP* gene in potato and pepper under darkness, low temperature NaCl, and PEG treatments

**Figure 8:**
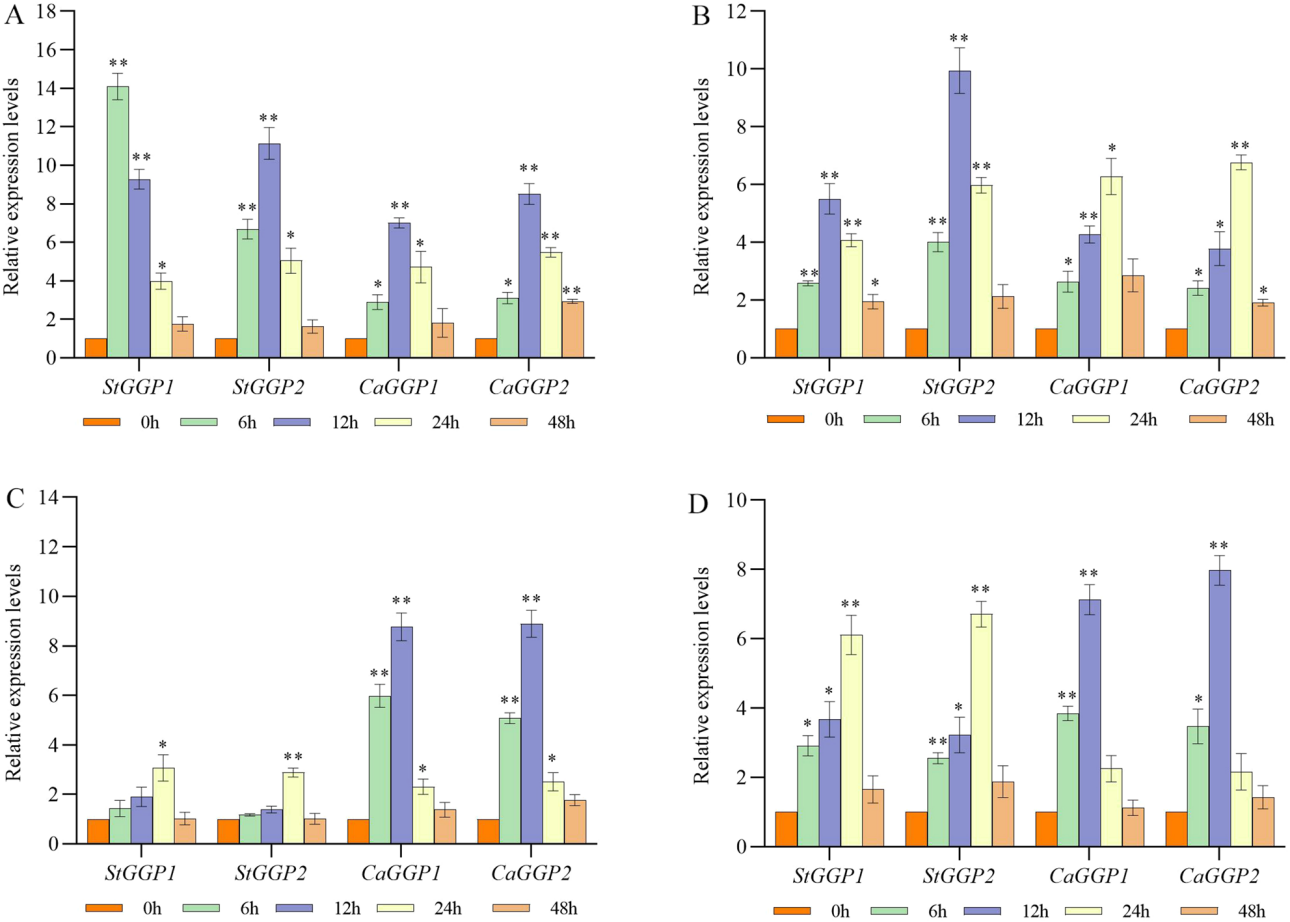
Relative expression levels of *StGGPs* and *CaGGPs* under different hormone treatments. (**A**) Dark stress. (**B**) Cold stress. (**C**) NaCl stress. (**D**) PEG stress. Expression values were normalized to the control (0 h) for each gene. An asterisk indicates a significant difference between the stress-treated and control groups. (*p* ≤ 0.05, **p ≤ 0.01, one-way ANOV A, Tukey’s test).

Under abiotic stress treatments, both *StGGPs* and *CaGGPs* genes were differentially up-regulated after a certain period of stress. Under the duress of darkness, the level of expression of *StGGP1* reached a maximum at 6 h and increased by a factor of 14 compared to the level at 0 h. However, the expression levels of *StGGP2*, *CaGG1* and *CaGG2* were gradually upregulated after dark stress treatment, and all of them reached the maximum at 24 h. Overall, the relative expression levels of the *StGGPs* were higher than those of the *CaGGPs* under the dark stress treatment (Fig. 8A). The expression levels of *StGGPs* and *CaGGPs* were gradually upregulated with time after cold stress treatment, with the difference that the expression levels of *StGGPs* reached the highest after 12 h, while those of *CaGGPs* reached the highest after 24 h (Fig. 8B). Under NaCl and PEG stress treatments, the expression levels of both *StGGPs* and *CaGGPs* were differentially up-regulated. Under both stress treatments, the expression levels of *StGGPs* peaked at 24 h, whereas *CaGGPs* peaked at 12 h, and the expression levels at 48 h were similar to those at 0 h (Fig. 8C. D).

## Discussions

AsA is found in a wide range of plant tissues and is a multifunctional metabolite involved in many physiological processes such as photosynthesis, cell wall biosynthesis, seed germination, flowering, fruit softening and ripening, post-harvest storage, and plant resistance to adverse environments^21^. The *GGP* gene has been identified in several species^13^. In this study, a more comprehensive and systematic analysis of the potato and pepper *GGP* gene families has been undertaken with the aid of bioinformatic techniques. Four *GGP* genes (two in potato and two in pepper) have been identified. Meanwhile, the selective evolutionary pressure indicated that there is a trend towards the purification of *GGP* members in both potato and pepper, and the trend towards the purification of *GGP* in potato is higher than that in pepper. Using a multi-species phylogenetic tree, we found that the *GGPs* of potato and pepper are more conserved in evolution than those of Arabidopsis, eggplant and tomato. In addition, most plant *GGP* genes have similar gene structures and motif patterns, this suggests that plant *GGP* genes have conserved functions^22^. The analysis of the physicochemical properties of the potato and pepper *GGP* gene family members showed that the protein sequences of the potato and pepper GGP families differ very little, with amino acid lengths ranging from 371aa to 440aa. With the exception of *CaGGP2*, which has an isoelectric point greater than 7, the isoelectric point of the rest proteins is less than 7. These results suggest that GGP proteins have a higher content of acidic amino acids. In addition, the instability indices of the potato and pepper GGP families are greater than 40. This indicates that potato and pepper GGPs are unstable proteins. Meanwhile, transmembrane structure analysis and signal peptide prediction indicated that potato and pepper *GGP* families do not have transmembrane structures as well as signal peptides. These results show the similarity between the *StGGPs* and the *CaGGPs* in terms of their physico–chemical properties as well as their gene structure. Previous study had also shown that most plant *GGP* genes had similar gene structures and motif patterns^23^. These similarities suggest that the *GGP* gene may play the same role in growing and developing potato and pepper.

*GGPs* have different patterns of expression in different plant tissues. Overexpression of kiwifruit *GGP* in tomato led to a six-fold increase in AsA content in red fruit^15^. In potato we found that the *StGGP1* is highly expressed in leaves. In addition, the expression levels of *StGGP1* are higher than those of *StGGP2* in all tissues except stamens. This leads us to speculate that *StGGP1* is the major gene for AsA synthesis in potato. However, in contrast to this, the *GGP* family of peppers has the lowest expression in the leaves and the highest expression in the fruits. Meanwhile, the expression of *CaGGPs* genes gradually decreases as the peppers ripened. In addition, the expression of tomato *GGP* was low in fruits, and the expression tended to decrease with fruit ripening^24^. Similarly, previous study showed that most of the 12 banana varieties contained more AsA in the unripe stage than in the ripe stage^16^.

Study has shown that AsA is involved in photosynthesis as an electron carrier or electron donor, and that the pathway for the biosynthesis of l-galactose is closely linked to photosynthesis^25^. AsA synthesis and *GGP* expression in *Arabidopsis thaliana* were induced by high light stress^26^. Similarly, analysis of the roles of the homeopathic components in our study showed that the potato ^GGP^ family respondes mainly to light-responsive components (seven) and hormone-responsive components (three), in addition to stress-responsive components. There are also three light-responsive elements in the *GGP* family of pepper. These results suggest that the *GGP* family plays a major role in light response. In subsequent experiments we found a significant up-regulation of both *StGGPs* as well as *CaGGPs* after dark treatment. This is further evidence that the *GGP* gene family plays an important role in the response to light. In addition, we found that *StGGP1* contains response elements that regulate the circadian rhythm. Meanwhile, previous study has shown that AsA content in leaves is two-fold higher during the day than at night, which may be related to circadian-regulated response elements^27^. Therefore, the potato and pepper *GGP* families may have an effect on AsA levels through participation in photosynthesis.

Previous study has shown that the overexpression of tomato *GGP* gene improved fruit quality and resistance^15^. Previous study has shown that the expression of the *GGP* gene in kiwifruit was induced by light and abiotic stress, and was significantly correlated with the concentration of AsA^28^. MeJA induces chemical defense in plants by stimulating the expression of plant defense genes. MeJA treatment increased the activity of the promoter, induced the expression of GGP in kiwifruit, and enhanced the AsA level^28^. ABA-responsive elements (ABRE) are also important for the enhancement of *GGP* transcript levels and GUS activities of *GGP* promoters. In both potatoes and pepper, MeJA-responsive elements were found. However, ABA response elements were found only in potato. Interestingly, in our experiments, after treatment with ABA and MeJA, the expression levels of both *StGGPs* and *CaGGPs* were significantly increased. This may be due to the fact that the ABA treatment regulates the expression of other genes in the pepper and thus promotes the expression of the *CaGGPs*. *GGP* plays an important role in protecting plants against chilling stress by maintaining the pool and redox state of ascorbate^29^. In addition, our study showed that *StGGPs* and *CaGGPs* may also have different responses to cold stress, salt stress and drought stress. Previous studies in rice, leek and lettuce have also shown that the *GGP* gene plays an important role under high light and abiotic stress conditions^30–32^. These results suggest that although potato and pepper are very different in both morphological and environmental characteristics, their *GGP* genes may play the same role under abiotic stress. However, more studies are needed to understand the specific functions of the *GGP* gene family in plant growth and response to abiotic stress.

## Conclusions

In conclusion, we identified four *GGP* genes in the whole genomes of potato and pepper, of which potato and pepper each contains two, and all of their members show a trend of purified selection. Meanwhile, there are strong homology and conservation of the *GGP* family in potato, pepper, tomato, Arabidopsis, tobacco and eggplant. The potato and pepper GGP genes have a genetic basis for responding to a wide range of hormones and stresses. *StGGPs* and *CaGGPs* were expressed at different levels in a variety of tissues, with *StGGP1* having the highest expression level in leaves, *StGGP2* the highest in stamens, and *CaGGPs* having the highest expression at Dev1. The expression patterns of *GGP* genes indicate tissue-specific roles across different plant species. In addition, *StGGPs* and *CaGGPs* may be involved in the mitigation of abiotic stress and hormonal responses. Meanwhile, *StGGPs* responded mainly to dark stress, whereas *CaGGPs* responded mainly to NaCl stress. Therefore, this study may provide further research on the involvement of the *GGPs* gene family in growth regulation and stress response in potato and pepper. It may also provide a theoretical basis for further exploration of the functions of plant *GGP* members.

## Materials and methods

### Identification of members of the *GGP* gene family in potato and pepper

The whole genome data and annotation files for potato and pepper were downloaded from NCBI (https://www.NCBI.nlm.nih.gov/). Membership information and protein sequences of the tomato *GGP* gene family were downloaded from Solanaceae Genomics Network, and the tomato database was used to search for identified members of the *GGP* gene family as template genes. The Blast Compare Two Seqs function of the TBtools software was used for a preliminary comparison to obtain the transcriptome IDs of the candidate members of the potato and pepper *GGP* gene families. The structural domains of the potato and pepper *GGP* nominees were viewed using the NCBI Web CD search tool, and the TBtools visualization of NCBI CDD patterns. Candidate proteins with GGP structural domains were members of the potato and chili pepper *GGP* gene families, and the genes corresponding to these proteins were named *StGGP* (potato) and *CaGGP* (pepper) genes.

### Chromosomal localization of the *GGP* gene family in potato and pepper

The chromosomal location distribution of the *GGP* gene was analyzed using the “gene location visualize from GTF/GFF” function of the TBtools software. The *GGP* genes for potatoes and pepper were renamed based on their location on the chromosome.

### Conserved motif and protein conserved domain analysis

The MEME website was used to conduct an online analysis of the shared conserved structural domains of potato and pepper *GGP* gene families. The structure of the potato and pepper *GGP* gene families was visualized using the TBtools “gene structure view (advanced) function”.

### *GGP* gene family multiple sequence comparison and phylogenetic tree construction

Potato and pepper *GGPs* were subjected to multiple sequence comparison analysis using DNAMAN Software. The GGP protein sequences of Arabidopsis, cabbage (*Brassica campestris*), apple (*Malus domestica cv. Gala*), and tomato were downloaded from the Arabidopsis, Brassica, NCBI, and Solanaceae databases, respectively. The evolutionary tree was constructed using the MEGA11 software, using the neighbor joining method, with the number of replicates set to 1000 and the rest of the options set to default values.

The evolutionary tree was then further modified using an online website (https://www.evolgenius.info//evolview/#mytrees/clcle/123).

### Prediction of the role of homeopathic elements of the *GGP* gene family

TBtools was used to extract data 2000 bp upstream of the potato and pepper *GGP* genes from the potato and pepper gene databases. The data was submitted to the PlantCARE database (http://bioinformatics.psb.ugent.be/webtools/plantcare/) for gene homeotic element (promoter) analysis and was visualized using TBtools.

### Analyzing protein structure and expression patterns

The Expasy online website (https://web.expasy.org/compute_pi/) was used to analyze the molecular weights, instability coefficients, isoelectric points and hydrophilicity of individual members of the potato and pepper *GGP* genes. The WoLF PSORT online website (https://wolfpsort.hgc.jp/) was used to analyze the subcellular localization of each member of the potato and pepper *GGPs*. The signal peptides of the GGP proteins of potatoes and peppers were predicted using SignalP (https://services.healthtech.dtu.dk/service.php?SignalP-5.0). The NPS online website (https://npsa-prabi.ibcp.fr/cgi-bin/npsa_automat.pl?page=npsa_sopma.html) was used to predict the secondary structure. The TMHMM online website (https://services.healthtech.dtu.dk/service.php?TMHMM-2.0) was used for transmembrane structural analysis. The UniPort online website (https://www.uniprot.org/) was used to extract expression data of the potato *GGP* family in different tissues. The pepper *GGP* expression profile was analyzed using the transcriptome data set obtained from the sequencing of the pepper genome (https://www.ncbi.nlm.nih.gov/geo/query/acc.cgi?acc=GSE45037) and visualized using TBtools^33^.

### Transcription analysis of the *GGP* gene under different abiotic stresses and hormonal conditions

The potato “Atlantic” seedlings were selected, and the medium (4.43 g. L^−1^ MS + 1.0 mg. L^−1^NAA (naphthalene acetic acid) + 0.2 mg. L^−1^6-BA (6-benzylaminopurine) + 30 g. L^−1^ sucrose + 6.5 g. L^−1^ agar) was used as the basic culture medium. The cultures were incubated in an artificial climate chamber at 23 ℃, 16 h of light/16 ℃, 8 h of darkness. Potato seedlings with good growth similarity and free of contamination were selected for subsequent treatment after 35 days of incubation. For NaCl, ABA, Methyl jasmonate (MeJA), and PEG 6000 treatments, seedlings were transplanted into 1/2 Hoagland nutrient solution containing NaCl (200 mM), ABA (100 µM), MeJA (50 µM), and PEG 6000 (10%) and incubated for 0, 6, 12, 24, and 48 h. The treatment concentrations used were obtained based on pre-experiments. For the cold treatments, the seedlings were placed in 1/2 Hoagland nutrient solution and in a refrigerator (Qingdao Haier Specialty Appliances Co., Ltd., Qingdao, China) at 4 ℃ for 0, 6, 12, 24, and 48 h. For the dark treatment, potato seedlings were treated by placing them in black, airtight, breathable boxes. Leaf samples were collected for qRT-PCR experiments after treatment at 0, 6, 12, 24, and 48 h.

The pepper variety “Qiang feng 101”, provided by the Vegetable Breeding Laboratory, College of Horticulture, Gansu Agricultural University, was used as the test material for this experiment. The pepper seeds were soaked and allowed to germinate, then sown in hollow trays. The seedlings were grown under day/night temperatures of 25 °C/20 °C, relative humidity of about 60%, 14 h of light and 10 h of darkness. When the pepper seedlings had six true leaves, they were treated in the same way as potatoes.

The collected samples were immediately frozen with liquid nitrogen and stored in a vertical ultra-low-temperature refrigerator at −80 ℃ (Qingdao Haier Special Electric Appliance Co., Ltd., Qingdao, China). Each treatment contained three biological replicates.

### RNA extraction and qRT-PCR fluorescence quantification

Total RNA was extracted from the samples using TRIzol reagent (Invitrogen, Carlsbad, CA, USA), taking advantage of the FastQuant First Strand cDNA Synthesis Kit (Tiainen, Beijing, China) to synthesize cDNA (The reaction system was 2 µL RNA, 2 µL 5 × Evo M-MLV Reagent Premix and 6 µL ddH2O). Dilute the cDNA concentration to 500 ng. µL^−1^. These reactions were carried out under the following conditions: 37 °C for 15 min, 85 °C for 5 s, and finally ending at 4 °C. LightCycler 480 real-time PCR system (Roche Applied Science, Penzberg, Germany) and SYBR Green Premix Pro Taq HS Premix kit were used for qRT-PCR. The reaction system was 2 × SYBR Green Pro Taq HS Premix 10 µL, primer (The concentration was 10 μ mol. L^−1^) F 0.4 μL, primer R 0.4 μL, cDNA 2 μL, and ddH2O 7.2 μL. The primers used in the qRT-PCR were designed using Primer 5.0 and their specificity was confirmed by melting curve analysis. PCR cycling was performed as follows: 2 min at 95 °C followed by 39 rounds of 5 s at 95 °C, 30 s at the optimal annealing temperature and finally, one cycle of 5 s at 65 °C. A melting curve (65–95 °C; at increments of 0.5 °C) was generated to verify the specificity of primer amplification. Three replicates of each tissue sample were used to monitor possible sampling and experimental errors. The efficiency of each primer pair was determined by generating a standard curve using sequence dilutions of the cDNA. ct values were within the linear amplification range to ensure the reliability of the data. The potato and pepper ACTIN genes were used to normalize relative expression levels, respectively. The specific primer sequences are shown in Table S1. The qRT-PCR data were analyzed using the 2^−ΔΔCt^ calculation method^34^.

### Data statistics and analysis

All experiments were conducted with at least three independents biological replicates, and all reported data were presented as mean ± standard deviation (SD). Statistical analysis was performed using SPSS statistical software 22.0 (SPPS Inc. Chicago, IL, USA). Multiple comparisons were performed with the Tukey’s test when one-way ANOVA showed a significant effect. (*p ≤ 0.05, **p ≤ 0.01). Means ± SD was based on three replicates. Graphs were constructed using GraphPad prism 9.0.0 (GraphPad Software, San Diego, CA).

### Supplementary Information

Supplementary Information.

## Data Availability

All data generated or analyzed during this study are included in this article. Data is provided within the manuscript or supplementary information files.
